# Influence of conjugated linoleic acid on the porcine immune response and morbidity: a randomized controlled trial

**DOI:** 10.1186/1476-511X-8-22

**Published:** 2009-06-22

**Authors:** Tomaz Malovrh, Lidija Kompan, Polona Juntes, Branka Wraber, Alenka Spindler-Vesel, Drago Kompan

**Affiliations:** 1 University Medical Centre Ljubljana, Ljubljana, Slovenia; 2 Insitute of Oncology, Ljubljana, Slovenia; 3 Veterinary Faculty, University of Ljubljana, Ljubljana, Slovenia; 4 Medical Faculty, University of Ljubljana, Ljubljana, Slovenia; 5 Biotechnical Faculty, Zootechnical Department, University of Ljubljana, Ljubljana, Slovenia

## Abstract

**Background:**

Conjugated linoleic acid (CLA) has diverse influences on the immune response in different experimental models. In the present study we investigated the effect of CLA feeding on inflammatory and immune responses in a piglet model. We studied the duration of this effect and possible detrimental effects of CLA feeding. After 12 weeks of CLA and control supplementation and washout, animals were sacrificed and parenchymal organs were histologically examined.

**Results:**

In activated peripheral mononuclear cells interferon-γ was significantly (p = 0.008) lower in the CLA group by the end of the feeding period. This effect disappeared as soon as supplementation was stopped. No differences were found in the tumour necrosis factor-α, interleukin-10 production, serum immunoglobulin-G levels and fat infiltration of the liver, except that fat storage cell infiltration was significantly (p < 0.04) higher in the CLA-fed group. The effect of time for interferon-γ, interleukin-10 and immunoglobulin-G levels was statistically significant.

**Conclusion:**

At the end of the feeding period the interferon-γ response was depressed. However, the maturation of the piglet immune system in our young pig model probably outweighs the impact of CLA feeding on the immune response, even though liver fat storage cell infiltration, which plays an important role in liver regeneration, increased during CLA feeding of the piglets.

## Background

Conjugated linoleic acids (CLA) are naturally occurring fatty acids found in dairy products and the meat of ruminants. CLA isomers are formed in the rumen of ruminants as intermediates in the hydrogenation of linoleic acid to vaccenic acid, with the cis-9, trans-11 CLA isomer (c9, t11-CLA) accounting for at least 90% of the possible isomers. Polyunsaturated fatty acids are known to influence the immune system. CLA, for instance, stimulates CD8+ lymphocytes and thymocytes. CLA also decreases inflammatory tissue damage by modifying cell membranes and influencing eicosanoid production and cell signalling events [1,2]. However, different isomers have different effects. Differences also exist among mammalian species, with mice being the most sensitive [3,4]. Similarities between pigs and humans makes the pig a frequently used model for CLA investigations [5-8]. In the pig model, it was confirmed that dietary CLA prevents the elevation of the proinflammatory cytokines interleukin-6 (IL-6) and tumor necrosis factor-α (TNF-α), induced by the lipopolysaccharide (LPS) challenge, but enhanced the expression of interleukin-10 (IL-10) in the spleen and thymus [9]. IL-10-mediated effects were dependent on the inhibition of nuclear factor-κB (NF-κB) activation and it was demonstrated that c9, t11-CLA enhanced this effect [10].

It has also been proven that CLA enhances cellular immunity by modulating phenotype and effector functions of CD8+ cells [6,8]. The CD4+ Th1 response has been studied in CLA-fed and porcine circovirus type 2 infected pigs, and found to suppress interferon-γ (IFN-γ) production [8]. Yet, on *in vitro *stimulated Jurkat cells, analysis of IL-2 and IFN-γ transcript levels showed increased expression of both cytokines on CLA-treated cells [11]. After feeding was completed, the immunomodulatory effect of dietary CLA on the immune cell phenotype persisted 67 days, compared to 25 days for effector functions [6].

However, CLA feeding seems to affect lipid accumulation in different parechymal organs. Thus CLA affects accumulation of lipids [12] and interconversion of fatty acids in the mouse liver [13], but reduces hepatic steatosis in CLA fed rats [14].

Because of these diverse influences of CLA on the immune response and metabolism, we investigated the effect of CLA feeding on the modulation of the inflammatory and immune responses in a pig model in the present study. The peripheral blood mononuclear cells (PBMC) of CLA and non-CLA supplemented pigs were isolated and activated either by polyclonal activators (to assess the modulatory effect of CLA feeding on IFN-γ and IL-10 production) or by LPS to assess the modulation of TNF-α. The IFN-γ and IL-10 secretions were assessed in our own model of *in vitro *cytokine production designed for monitoring the subtle changes in cytokine responses occurring *in vivo *due to the pathological processes and/or immuno-interventions in humans [15-17]. During the experiment immunoglobulin-G (IgG) levels were measured. After the feeding was completed the duration of any possible immunomodulatory effect was also tested. Finally, the effect of CLA feeding on lipid metabolism was evaluated by the changes of the lipid content in the liver and kidneys.

## Methods

### Experimental animals, diets and design of the experiment

Twelve piglets of the Slovenian Landrace breed, weaned at the age of nineteen days were used in the experiment. The animals were randomly assigned into experimental and control groups and housed in individual cages. Each group of six piglets consisted of three females and three male castrates. Initially they were fed "ad libitum" until the age of 4 weeks with an experimental feed which consisted of wheat (60%), maize (8%), barley (5%), extracted soya bean meal (10%), fish meal (3%) and other special protein concentrates (10%). The feed contained 13.5 MJ of metabolic energy (ME), 18% of crude proteins with 12.5 g of Lysine per kg of feed, 2.95% crude fiber and 3.7% crude fats. The piglets were then weighted, the average weight at the beginning of supplementation was found to be 6.66 kg and the average maintenance energy (MaE) was calculated accordingly:



(MaE = maintenance energy in kJ/day, BM = body mass in kg).

During the next phase of the experiment the piglets were fed 1.8 times maintenance energy starting at an average 400 g of experimental feed per day per piglet. The piglets were weighed every week and maintenance energy was assigned individually at that time using the before mentioned MaE equation. Thus, between the 41 and 47 days of age, the piglets received on average 560 g of feed daily, between the ages of 48 and 54 days 630 g per day, between the ages of 55 and 61 days 710 g per day, between 62 and 68 days of age 800 g per day, and between the ages of 69 and 76 days 870 g per day.

The experimental group of piglets received a 1.2% supplement of a 50:50 mixture of c9, t11-CLA and trans-10, cis-12 CLA (t10, c12-CLA) isomers (Larodan ABLimhamnsgårdens alle 9, S-216 16 Malmö Sweden), i.e. 1.2 g of pure CLA per 100 g of feed. Because the purity of CLA was only 80%, the CLA supplement was actually 1.5% (1.5 g per 100 g of feed). The control group received 1.5% sunflower oil. After 5 weeks of supplementation, CLA isomer feeding was stopped. In the last phase of the experiment, the piglets were kept individually and fed on the same diet for the remaining seven weeks without any supplement. Twelve weeks after the start of supplementation all remaining piglets were humanely sacrificed and necropsies were performed to evaluate possible gross pathology lesions. Samples of organs and tissues for further post-mortem analyses were collected at the same time.

### Isolation, preparation, *ex vivo *challenging of PBMC and IgG determination

Blood samples were taken every second week starting at day 0 during CLA feeding and after the supplementation ended. Peripheral blood mononuclear cells (PBMC) were then isolated and treated for cytokine responses as previously described for human PBMC [15-19]. Piglet mononuclear cells from each sample of venous blood with ethylenediaminetetracetic acid (EDTA) were isolated by Ficoll-Paque (Phadia, Sweden) density gradient centrifugation. The cells were suspended in RPMI 1640 supplemented with 100 U/ml penicillin, 100 μg/ml streptomycin, 2 mM L-glutamine and 5% heat-inactivated foetal calf serum (Sigma, St. Louis, Missouri, USA) and plated in 24-well cell culture plates (T grade, NUNC, Roskilde, Denmark) to a final concentration of 6.66 × 10^5^/ml. To establish the TNF-α response to the LPS challenge, PBMCs were incubated for 18 hours with LPS (from E. coli, 0111:B4; Sigma, St. Louis, Missouri, USA), with a final culture concentration of 10 ng/ml. Evaluation of the IFN-γ response was performed by simultaneous polyclonal activation by phorbol 12-myristat 13-acetate (PMA), with a final concentration of 3.33 ng/ml, and ionomycin (IONO), with a final concentration of 500 nM. Plates were then incubated for 40 hours. Plates with LPS and plates with IONO&PMA were incubated at 37°C, in a 5% CO_2 _atmosphere and 95% humidity. The cell-free supernatants were collected at 18 and 40 hours, respectively. Supernatant samples were stored at -70°C before being evaluated for three cytokines using commercially available ELISA kits for measuring porcine TNF-α, IFN-γ (Pierce – Endogen, Rockford, IL, USA) and IL-10 (R&D Systems, Minneapolis, MN, USA).

Total IgG was determined by serum dilution 1:32000 and than measured using an ELISA kit (Abbott Co., Chicago, IL., USA).

### Pathoanatomical and histopathological examination

Whole body necropsies were performed on pigs from the control group and those from the CLA-fed experimental group. Parenchymal organs were weighted, examined and samples of selected parenchymal organs collected for histopathological examination. The main target organ for the study was the liver, due to its role in the metabolism of lipids and its excretory function. During the necropsy samples were taken from the four liver lobes (left and right, lateral and medial lobes) and from the kidneys. Tissue blocks for histopathology were fixed in 4% formalin buffered with Di-sodiumhydrogenphosphate, Sodiumdihydrogenphosphate and embedded in paraffin. Four μm thick tissue sections were stained with haematoxylin and eosin (HE) for examination by light microscopy, while 10 μm thick frozen sections of formalin-fixed liver and kidneys were stained for lipid content with Sudan III [20].

The distribution pattern within liver lobules (periportal, midzonal, centrolobular) and localization within hepatocytes and fat-storing cells was determined for every liver lobe. Quantification under light microscopy was subjective and the amount of lipid in liver and kidney was evaluated using light microscopy and the following descriptive scale: 0 – negative, 1 – barely visible, 2 – mild, 3 – moderate, 4 – strong, 5 – very strong.

The ethics of the study protocol was approved by the Slovenian Veterinary Administration, Parmova ulica 53, Ljubljana, Slovenia.

### Statistical analysis

Descriptive statistics were calculated for all the observed parameters. Two-way mixed analysis of variance (ANOVA) was used to analyse the data with sampling day as within-subject factor (6 levels) and group as between-subjects factor (2 levels), whereby the Greenhouse-Geisser correction was applied when Mauchy's test indicated significant departure from the sphericity assumption. It should be noted that if instead of ANOVA, analysis of covariance (ANCOVA) was used with day 0 as covariate and only the remaining 5 sampling days as levels of the time factor, the same conclusions were reached. Additionally, independent sample t-tests were performed to asses the difference in means between the two groups on each sampling day except Day 0 (when any difference claimed between the groups would be a Type I error by definition) [21]. The mean liver infiltration was calculated for each individual type of infiltration, after which a t-test was performed to compare the control and CLA-fed groups. A similar procedure was performed for the evaluation of kidney tubular cell fat infiltration. A P ≤ 0.05 value was considered statistically significant. All statistical analyses were performed using SPSS for Windows software (version 15.0.1.1., Chicago, IL, 2007).

## Results

### Immunological examination

ANOVA showed no significant effect of group or interaction for IFN-γ, TNF-α, IL-10 or IgG (Table 1, 2, 3, and 4). The effect of time was statistically significant for IFN-γ, IL-10 and IgG, thus indicating differences in response levels between the sampling days for these parameters, whereby the time-course did not differ significantly between the groups.

**Table 1:** The effect of conjugated linoleic acid (CLA) on the porcine IFN-γ response.

**Parameter and results of ANOVA**		**Day**	**Control group**	**CLA group**
**IFN-γ **(pg/ml)		0	3911 (4030)	6896 (3398)
		14	9519 (8177)	5639 (4561)
**Effect of**	**P**	28	7356 (3489)	9132 (3967)
Group	0.177	42**^#^**	12838 (6830)	3345 (1726)
Time	0.047*	56	10875 (6034)	14624 (6774)
Interaction	0.145	70	16873 (12310)	10660 (6501)

Descriptive statistics (mean and SD) and results of ANOVA, *P ≤ 0.05

^# ^The comparison between groups that showed significant difference using t-test (p = 0.008) and after Bonferroni correction.

**Table 2:** The effect of conjugated linoleic acid (CLA) on the porcine TNF-α response.

**Parameter and results of ANOVA**		**Day**	**Control group**	**CLA group**
**TNF-α **(pg/ml)		**0**	47.3 (79.9)	43.5 (45.5)
		**14**	15.3 (6.1)	93.0 (98.2)
**Effect of**	**P**	**28**	32.2 (17.5)	54.4 (49.3)
Group	0.105	**42**	53.9 (19.6)	78.0 (66.9)
Time	0.367	**56**	54.2 (31.8)	34.3 (24.9)
Interaction	0.417	**70**	87.6 (60.6)	98.6 (70.5)

Descriptive statistics (mean and SD) and results of ANOVA

**Table 3:** The effect of conjugated linoleic acid (CLA) on the porcine IL-10 response.

**Parameter and results of ANOVA**		**Day**	**Control group**	**CLA group**
**IL-10 **(pg/ml)		0	9.1 (5.7)	14.6 (4.5)
		14	12.2 (7.1)	10.68 (6.9)
**Effect of**	**P**	28	9.20 (8.2)	15.47 (6.5)
Group	0.247	42	13.8 (7.7)	13.73 (4.6)
Time	0.045*	56	11.0 (7.4)	19.04 (7.8)
Interaction	0.489	70	20.1 (3.8)	22.6 (4.8)

Descriptive statistics (mean and SD) and results of ANOVA, *P ≤ 0.05

**Table 4:** The effect of conjugated linoleic acid (CLA) on the porcine serum IgG values.

**Parameter and results of ANOVA**		**Day**	**Control group**	**CLA group**
**IgG **(mg/ml)		0	0.08 (0.03)	0.06 (0.03)
		14	0.09 (0.03)	0.08 (0.02)
**Effect of**	**P**	28	0.11 (0.03)	0.14 (0.03)
Group	0.914	42	0.08 (0.02)	0.08 (0.02)
Time	0.001*	56	0.19 (0.04)	0.23 (0.05)
Interaction	0.516	70	0.23 (0.04)	0.24 (0.03)

Descriptive statistics (mean and SD) and results of ANOVA, *P ≤ 0.05

Comparison between the control and CLA-fed groups at each sampling day yielded a single P-value below 0.05, namely for the IFN-γ response to polyclonal activation by IONO&PMA by the end of the feeding period (at day 42), when the measured level of IFN-γ was significantly lower in the CLA-fed group (p = 0.008). This difference remains significant even when adjusted for multiple tests using Bonferroni correction (i.e., multiplied by 5).

### Pathoanatomical and histopathological examination

Necropsy revealed no differences between the two groups. These findings were confirmed histopathologically.

Histopathology of the lungs revealed some chronic, mostly organised bronchointerstitial pneumonic lesions in 5 pigs from the control and 6 from the CLA-fed group. Acute pneumonic infiltrates were also identified in one pig in the control and two in the CLA-fed group.

The liver was examined more closely. No significant difference in liver weight, expressed as a percentage of body weight, was found between the groups, with an average of 1.73% in the control and 1.85% in the CLA-fed group (p = 0.131).

Evaluation of the different distributions of liver fat infiltration revealed no significant differences between the two groups, while the mean score of fat storage cell fat infiltration was significantly higher in the CLA-fed group (p < 0.04) (Table 5).

**Table 5:** The effect of conjugated linoleic acid (CLA) on the liver fat infiltration.

**Distribution of fat infiltration**	**Mean fat infiltration score**		**P**
	**Control group**	**CLA group**	
Periportal	1.92 (0.92)	1.41 (1.14)	0.403
Midzonal	1.17 (0.98)	0.75 (1.07)	0.483
Centrolobular	1.17 (0.98)	0.75 (1.07)	0.483
Fat storage cells*	0.0 (0.0)	0.59 (0.47)	<0.04

Descriptive statistics (mean and SD), *P ≤ 0.05

Evaluation of kidney tubular cell fat infiltration also showed no significant difference between the groups, with a mean score of 2.58 in the control and 1.07 in the CLA-fed group (p = 0.072).

## Discussion

In the present study, a notably decreased IFN-γ response to polyclonal activation by IONO&PMA was found in the CLA-fed group at the end of the feeding period. This effect disappeared when the next post-feeding sample was taken. No differences were found in the TNF-α and IL-10 response to LPS and IONO&PMA *ex vivo *challenge between CLA and non-CLA-fed pigs, as well as in serum IgG levels. The *ex vivo *LPS and IONO&PMA challenge, which we used in this study was, to our knowledge, also the first ever performed on pigs in studies of the immunological influence of CLA. No difference in post-mortem infection rate was found between our two groups of animals.

We noticed a statistically significant effect of time for IFN-γ, IL-10 and IgG in both groups, thus indicating differences in response levels between the sampling days for these parameters. This finding, however, is probably a consequence of maturation of the piglet immune system, which seems not to be influenced by CLA feeding, as it does not affect the observed difference between the control group and the CLA-fed group on day 42. The fact that no statistically significant effect of group or interaction was found by ANOVA for any parameter (not even for IFN-γ), can be attributed to the small sample size and relatively large within-group variability, due to which the between-group comparisons at individual days provided valid additional information.

Some other studies on the immunological response to CLA feeding have yielded a broad range of different results. An CLA-fed porcine model was tested for cellular immunity *in vivo *by vaccination and/or infection challenge, CD4+ and CD8+ T cells isolated from virally infected pigs were studied for IFN-γ production on *ex vivo *re-stimulation: CLA was found to suppressed IFN-γ production in CD4+ but not in CD8+ T cells in these experiments [5-8]. Fourteen day old piglets, in the experiment which lasted 35 days, were used and in these studies the importance of pig immune system maturation was not taken into consideration [5]. Bassaganya-Riera *et al*. maintain that up to 35 days of dietary CLA supplementation effects immature thymocites and only protracted feeding modulates peripheral blood T cells. We kept our CLA feeding for 35 days, after which we found transiently depressed levels of IFN-γ, which is in accordance with Bassaganya-Riera's *et al*. finding, that IFN-γ production is suppressed in CD4+ cells  [5,8]. Changhua *et al*. used a 28 day old pig model to study the acute macrophage response. They supplemented CLA for only 14 days and found inhibitory actions of CLA on IL-1β, IL-6, TNF-α and increased IL-10 levels after *in vivo *LPS stimulation in pigs, which were attributable mainly to t10, c12-CLA [9]. In contrast to Changhua *et al*., our LPS stimulation was carried out *ex vivo*, and it is interesting that the effect on TNF-α in our porcine experiment was the same as that in humans stimulated *ex vivo*, where CLA had no effects on *ex vivo *LPS-stimulated cytokine production [22]. In contrast to our results they also found an increase in IgG concentration of colostrums during dietary supplementation with 0.5% CLA to sows during late gestation [23].

In rodents CLA has been demonstrated to suppress production of proinflammatory cytokines, but individual isomer supplementation also makes a difference [24,25]. Thus, lymphocytes isolated from c9, t11-CLA-fed mice produced more TNF-α than the control group. Yamasaki *et al*. found that, compared with t10, c12-CLA, c9, t11-CLA significantly stimulated TNF-α production in spleen lymphocytes of mice *in vivo *[26]. On the contrary, Yang and Cook ascribed inhibition of LPS-induced TNF-α production in mice to c9, t11-CLA [2]. In terms of CLA influence on immunoglobulin production, mice fed t10, c12-CLA produced more immunoglobulin A (IgA) and IgM but not IgG [26]. However, omega-3 fatty acids have also been found to influence IgG, but not IgA and IgM production in rats [27].

In humans, daily consumption of 3 g of the c9, t11-CLA or t10, c12-CLA isomer did not affect LPS-stimulated cytokine production [22]. Likewise, CLA had no effects on *ex vivo *cytokine production, but CLA supplementation did reduce mitogen-induced activation of T lymphocytes [28]. *In vitro *experiments on human cells demonstrated that the effects of CLA on cellular prostanoid formation can be either inhibitory or stimulatory [29,30]. On the other hand, CLA decreased TNF-α and IFN-γ production after 12 weeks of feeding in humans in another study [31]. Different influences of individual CLA isomers on immunoglobulin subclasses were also observed in humans [32,33]. This leads to the impression that CLA might have multiple, complex immunological effects in humans and presumably also in pigs [30].

Thus, results of studies on the immunological role of CLA are not uniform. It is therefore obvious that the immunological effect of CLA depends not only on the species, but also on the gender, the CLA isomer used, duration of feeding, whether the cells are in a resting or stimulated state [1,30,31,34] and on *in vivo *versus *in vitro *immunological stimulation.

Adverse side effects, such as insulin resistance, hyperinsulinaemia, inflammatory changes in adipocytes and liver steatosis, have been reported mostly in mice, with some evidence existing in humans. These effects are probably due to t10, c12-CLA [35]. Our post-mortem investigation of organs, with special emphasis on the lipid content in the liver, failed to reveal any evidence of these effects. However, morphological evaluation of the lipid distribution pattern and semiquantitative evaluation of the lipid amount showed slightly higher, though insignificant, amounts of fat in the liver of the control group. On the other hand, a significant difference was found in the lipid content of fat storing cells, where higher amounts were found in the CLA-fed group. Fat storing cells, also called hepatic stellate cells, are involved in the metabolism of retinol and play an important role in liver regeneration [36]. Hepatic stellate cells in arctic animals store 20–100 times the levels of retinol-palmitat lipid droplets as found in humans and rats [37]. If we anticipate that Arctic animals consume more unsaturated fats, this could be in agreement with our findings. Thus, CLA feeding probably isn't detrimental to the pig liver as it has been shown to be in some rodent models [13,38].

## Conclusion

In our CLA-fed pigs, transitory immunomodulatory effects were observed at the end of the feeding period with a depression of the IFN-γ response. However, CLA feeding had no effects on *ex vivo *LPS stimulation. Moreover, our results indicate that the maturation of a piglet's immune system probably outweighs the impact of CLA feeding on their immune response. This should, therefore, be taken into account by studies on the immune response of young piglets. Our study also shows that the brief CLA feeding of pigs has no other pathomorphological influence besides that on hepatic fat storage cell fat infiltration, which seems to have a hepatoprotective effect.

## Competing interests

The authors declare that they have no competing interests.

## Authors' contributions

All authors read and approved the final manuscript.

TM wrote the study, analysis and interpretation of data, LK designed and wrote the study, PJ has done pathoanatomical analysis, BW has done immunological analysis, ASV collection of data and immunological analysis, DK conception, design and final approval.
